# Conventional protein kinase C in the brain: 40 years later

**DOI:** 10.1042/NS20160005

**Published:** 2017-04-10

**Authors:** Julia A. Callender, Alexandra C. Newton

**Affiliations:** 1 Department of Pharmacology, University of California, San Diego, La Jolla, CA 92093-0721, U.S.A.; 2 Biomedical Sciences Graduate Program, University of California, San Diego, La Jolla, CA 92093-0721, U.S.A.

**Keywords:** Alzheimer's disease, neurodegeneration, posttranslational modification, protein kinase C

## Abstract

Protein kinase C (PKC) is a family of enzymes whose members transduce a large variety of cellular signals instigated by the receptor-mediated hydrolysis of membrane phospholipids. While PKC has been widely implicated in the pathology of diseases affecting all areas of physiology including cancer, diabetes, and heart disease—it was discovered, and initially characterized, in the brain. PKC plays a key role in controlling the balance between cell survival and cell death. Its loss of function is generally associated with cancer, whereas its enhanced activity is associated with neurodegeneration. This review presents an overview of signaling by diacylglycerol (DG)-dependent PKC isozymes in the brain, and focuses on the role of the Ca^2+^-sensitive conventional PKC isozymes in neurodegeneration.

## Introduction

Protein kinase C (PKC) was discovered in the late 1970s by Nishizuka and colleagues as the pro-enzyme of a constitutively active kinase they had purified from bovine brain [1,2]. The originally purified enzyme was termed protein kinase M (PKM), as the only cofactor required for activity was Mg^2+^ [2]. Subsequent studies revealed that PKM was a proteolytic product of a parent pro-enzyme whose activity was stimulated by Ca^2+^ and the particulate fraction of brain extracts; they named the pro-enzyme PKC for its activation by the second messenger Ca^2+^ [3]. In a series of classic biochemical studies involving adding back components of brain extracts, the ‘activators’ of the pro-enzyme were identified as phospholipids, notably phosphatidylserine (PS) [3], and a ‘trace impurity’ later identified as diacylglycerol (DG) [4]. The discovery that PKC (whose official name was now Ca^2+^-activated, phospholipid-dependent kinase) was directly activated by DG provided the long-sought effector for the phospholipid hydrolysis that earlier studies by Hokin and Hokin had shown was provoked by cholinergic stimulation [5]. But the discovery that catapulted PKC to the forefront of signaling was its identification as a receptor for the tumor promoting phorbol esters [6], a finding made possible by the synthesis of relatively water soluble phorbol esters, notably phorbol dibutyrate (PDBu) by Blumberg and colleagues [7]. This diverted studies of PKC from the brain to understanding its role in cancer [8]. Yet 30+ years of clinical trials for cancer using PKC inhibitors not only failed [9], but in some cases worsened patient outcome [10]. It took analysis of cancer-associated mutations in PKC to reveal that the multiple isozymes in this family generally function to suppress survival signaling [11], so therapies for cancer should focus on restoring rather than inhibiting activity. In striking contrast with the loss-of-function mutations in cancer, gain-of-function mutations are associated with neurodegenerative diseases, including Alzheimer's disease (AD) [12]. Indeed, PKC isozymes play roles in a variety of brain pathophysiologies, including alcoholism, opiate addiction, epilepsy, stroke, and glioblastoma [13–17]. Here, we provide an overview on PKC and the function of the Ca^2+^/DG-regulated isozymes in neuronal signaling and pathologies.

## Regulation of PKC activity

**Figure 1:**
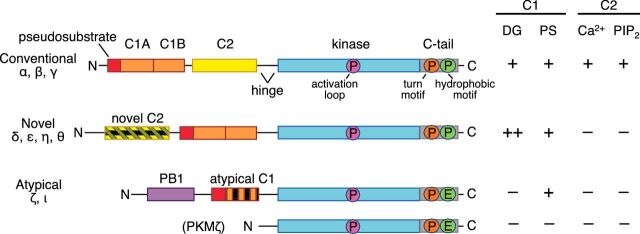
Domain composition of PKC family members PKC isozymes are classified into one of three subfamilies based on their domain composition (left), which in turn dictates their second messenger and cofactor sensitivity (right). The N-terminal regulatory moiety contains the autoinhibitory pseudosubstrate segment (red), the tandem DG-binding C1 domains (orange), and the Ca^2+^-binding C2 domain (yellow). The C2 domain in novel PKC isozymes and the C1 domain in atypical PKC isozymes are non-ligand binding variants (striped). Novel PKC isozymes are able to respond to increases in DG alone because their C1B domain binds this ligand with two orders of magnitude higher affinity than that of conventional PKC isozymes, whose cellular activation depends on signals that elevate both Ca^2+^ and DG. Atypical PKC isozymes have a PB1 domain (purple) that mediates binding to protein scaffolds. The C-terminal kinase moiety contains the catalytic domain that has a priming phosphorylation site by PDK-1 (pink) and a C-terminal tail that is phosphorylated at the turn motif (orange) and hydrophobic motif (green); atypical PKC isozymes have a Glu at the phosphoacceptor site of the hydrophobic motif. Also shown is a brain-specific splice variant of the kinase moiety of PKCζ and PKMζ.

The PKC family is encoded by nine genes whose protein products are organized into three subfamilies based on their cofactor dependence: conventional PKC isozymes (α, the alternatively spliced βI and βII, and γ) are activated by DG and Ca^2+^, novel PKC isozymes (δ, ε, θ, and η) are activated by DG, and atypical PKC isozymes (ζ, ι) are regulated by protein scaffolds (Figure 1) [18,19]. In addition, a splice variant of PKCζ lacking the regulatory moiety is expressed in the brain; it is named PKMζ in reference to the constitutively active kinase moiety PKM described above [20].

Biochemical analyses by Nishizuka's group originally showed that PKC activity is higher in brain than in any other tissue examined, with particular enrichment in synaptosomal membrane fractions [21]. Subsequent immunohistochemical studies revealed strong expression of PKC in both neuronal and glial cells in different regions of the brain [22–26]. Isozyme-specific differences were later unveiled with the generation of isozyme-specific antibodies. For example, while the conventional PKCα is expressed in both glial and neuronal cells in multiple brain regions including the cerebral cortex and the basal ganglia, it is most highly expressed in the hippocampus. PKCβ expression is more limited to neurons, in multiple brain regions. The expression of PKCγ is restricted to neurons and is not normally found outside the brain [27]. All of the novel PKC isozymes are also enriched in brain tissues, and in addition to the PKCζ splice variant PKMζ whose expression is restricted to brain, the full-length atypical PKC isozymes are themselves highly expressed in the brain, although they are mostly found in neurons and not in glia.

All PKC isozymes are processed by a series of ordered phosphorylations and ordered conformational transitions to yield a signaling-competent enzyme that is maintained in an autoinhibited conformation until the correct second messengers are present [28–30]. Specifically, binding of a pseudosubstrate segment in the substrate-binding cavity of the kinase domain prevents activation in the absence of agonist. Agonist binding to the DG-sensing C1 domain and Ca^2+^-sensing C2 domain breaks intramolecular contacts to ‘open’ PKC and permit substrate phosphorylation. PKCα is the only DG-dependent isozyme with an identified C-terminal PDZ ligand, which it uses to engage in interactions with PDZ domain-containing scaffold proteins such as protein interacting with C kinase 1 (PICK1), postsynaptic density protein 95 (PSD95), and synapse-associated protein 97 (SAP97) [31,32]. For extensive reviews of PKC structure, function, regulation, and pharmacology, the reader is referred to these studies [8,28,33–35].

**Figure 2:**
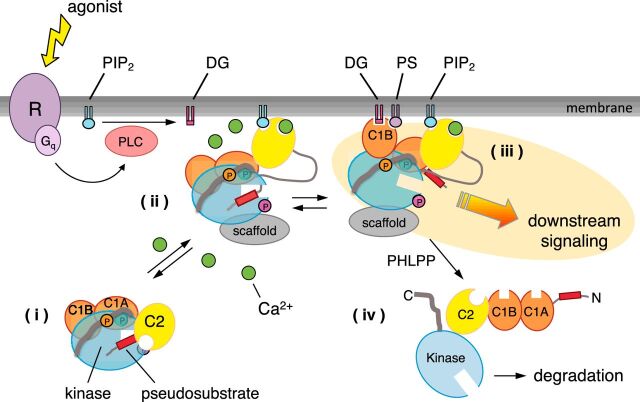
The priming, activation, and deactivation life cycle of a conventional PKC Newly synthesized PKC is processed via a series of tightly coupled phosphorylations (circles labeled ‘P’) to yield a matured species that localizes to the cytosol and is maintained in an autoinhibited conformation by intramolecular contacts between the kinase domain and the regulatory domains (species (i)). Agonist binding to Gq-coupled receptors (R) results in PLC-catalyzed hydrolysis of PIP_2_ to generate two second messengers: DG and, indirectly, Ca^2+^ mobilization. Ca^2+^ (green circle) binds the C2 domain, facilitating the bridging of the C2 domain to anionic phospholipids in the plasma membrane; specificity for the plasma membrane is mediated by binding of PIP_2_ to a distal surface on the C2 domain that is masked in the autoinhibited conformation of PKC (species (ii)). Subsequent binding of DG to the C1B domain, which also specifically binds PS, results in release of the pseudosubstrate from the substrate-binding cavity (species (iii)), allowing substrate phosphorylation and downstream signaling. Binding to protein scaffolds (gray), such as PSD95 for PKCα, can further constrain PKC signaling to specific intracellular locations. Prolonged membrane binding, as occurs upon treatment of cells with phorbol esters, results in dephosphorylation (species (iv)) and degradation of PKC, a process referred to as down-regulation. Novel PKC isozymes are similarly regulated except they do not have a Ca^2+^/plasma membrane sensing C2 domain and respond only to DG; their C1B domain binds DG with two orders of magnitude higher affinity than that of conventional PKC isozymes, so they respond to DG alone and localize primarily to DG-rich Golgi membranes.

Conventional and novel PKC isozymes move through a tightly regulated life cycle of signaling (Figure 2). Newly synthesized isozymes are in an open and degradation-sensitive conformation; they undergo a series of stabilizing phosphorylations at sites termed the activation loop, turn motif, and hydrophobic motif that lock the enzyme in an autoinhibited and stable conformation (Figure 2, species (i)) [18,19]. These phosphorylations—unlike those found in many other kinases—are constitutive and not agonist-evoked. The ‘matured’ PKC remains in the cytosol in its inactive, autoinhibited form [29,36]. Agonist-triggered, receptor-mediated activation of phospholipase C (PLC), typically via coupling with the G protein Gq, results in the generation of DG, the key allosteric activator of PKC [37]. Typically, the lipid hydrolyzed is phosphatidylinositol 4,5-bisphosphate (PIP_2_) resulting in release of the headgroup inositol trisphosphate (IP_3_) and Ca^2+^ mobilization [38]. Phosphoinositide signaling is particularly robust in neurons, with recent kinetic analysis revealing that PIP_2_ is resynthesized significantly more rapidly in these cells compared with electrically non-excitable cells [39]. Conventional PKC isozymes respond to both of these second messengers produced from PIP_2_ hydrolysis: first, Ca^2+^ binds the C2 domain to recruit PKC to the plasma membrane via bridging of the C2-bound Ca^2+^ to anionic phospholipids, such as PS, and PIP_2_ binding at a distal site that serves as a plasma membrane sensor (Figure 2, species (ii)). Once at the membrane, the enzyme binds its membrane-embedded ligand, DG, primarily by the C1B domain, which also specifically recognizes PS. Engagement of the C1B domain to the membrane provides the energy to release the autoinhibitory pseudosubstrate, yielding an open and active enzyme that can propagate downstream signaling (Figure 2, species (iii)). This membrane translocation is a hallmark of PKC activation, and movement to the membrane serves as a marker for the activation of the enzyme [40,41]. Binding to protein scaffolds, such as receptors for activated C kinase (RACKS) identified by Mochly-Rosen and co-workers in the early 1990s [42] or PDZ domain proteins, also play roles in localizing PKC and positioning it near substrates [43]. While a closed, autoinhibited PKC is resistant to dephosphorylation and degradation, an open PKC protein is now sensitive to phosphatases [44,45]. Consequently, prolonged activation of PKC results in its dephosphorylation and degradation (Figure 2, species (iv)), as is seen during chronic treatment with potent PKC activators such as phorbol esters and bryostatins, which cause the down-regulation of PKC [40,46]. Indeed overnight treatment with phorbol esters was a common way to deplete cells of PKC before the advent of siRNA and gene editing technologies. The precise regulation of each step of this pathway must be maintained for cellular homeostasis.

## PKC substrates in the brain

**Figure 3:**
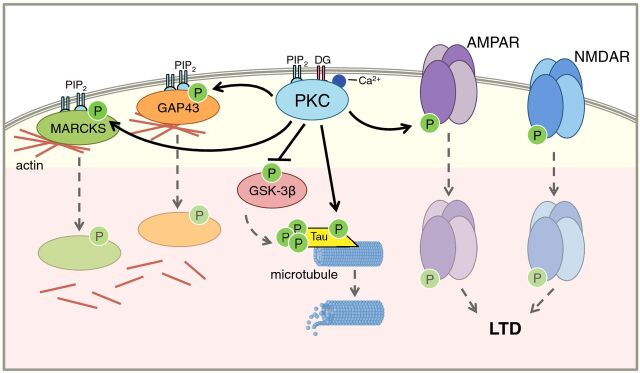
PKC substrates in the brain PKC phosphorylates many presynaptic and postsynaptic substrates; illustrative examples are depicted here on a generic membrane for ease of viewing. PKC (cyan) regulates the actin cytoskeleton (red lines) via its phosphorylation of MARCKS (green) and GAP43 (orange). Phosphorylation by PKC causes these proteins to translocate from the plasma membrane, where they facilitate actin polymerization, to the cytosol, thus promoting actin depolarization. PKC plays a role in microtubule (small blue circles) dynamics through its effects on the microtubule-associated protein tau (yellow). PKC both directly phosphorylates tau and indirectly causes the dephosphorylation of tau by phosphorylating and inactivating GSK-3β (pink). PKC regulates synaptic plasticity by regulating postsynaptic levels of AMPA receptor (purple) and NMDA receptor (blue). Phosphorylation by PKC causes internalization of these receptors, thus promoting LTD.

PKC phosphorylates a large variety of substrates and plays a role in many different signaling cascades. Here, we discuss some illustrative examples of both presynaptic and postsynaptic PKC substrates in the brain, which are summarized in Figure 3.

### Regulation of the cytoskeleton: MARCKS, GAP43, and tau

The myristoylated alanine-rich C-kinase substrate (MARCKS) was identified by Greengard and colleagues in 1982 as a protein highly enriched in brain that is heavily phosphorylated by PKC [47,48]. It has since become one of the most robust and well characterized read-outs of conventional and novel PKC signaling [49]. Its deletion in mice has revealed that it is required for mouse brain development and postnatal survival [50]. MARCKS binds to the plasma membrane via a myristoyl electrostatic switch, whereby the coordinated association of a hydrophobic N-terminal myristic acid with the bilayer and an adjacent basic segment with anionic phospholipid headgroups drives membrane association [51]. At the membrane, MARCKS facilitates the cross-linking of actin filaments [52]. Phosphorylation at multiple residues within the basic segment by PKC causes MARCKS to be released from the plasma membrane to the cytosol, thus promoting actin depolarization [51,53–57]. Growth-associated protein 43 (GAP43), required for neuronal development [58], is another conventional and novel PKC substrate that, similarly to MARCKS, normally localizes to the plasma membrane. Phosphorylation by PKC causes GAP43 to move away from the membrane [59], which promotes the disassociation of long actin filaments [60]. This phosphorylation also breaks GAP43’s interaction with calmodulin [61,62]. In addition to their roles in regulating the cytoskeleton, both MARCKS and GAP43 interact with and sequester PIP_2_ while at the plasma membrane. Release of these proteins from the membrane as a result of phosphorylation by PKC therefore leads to an increase in available PIP_2_ levels for other cellular processes such as endocytosis [63,64].

PKC also regulates microtubule dynamics through its effects on tau, a microtubule-associated protein that is highly enriched in neurons [65]. First, PKC directly phosphorylates tau in a non-pathological setting both *in vitro* and *in vivo* [66,67]. Second, PKC has been implicated in tau phosphorylation indirectly through its ability to phosphorylate and inactivate glycogen synthase kinase-3β (GSK-3β), thereby reducing tau phosphorylation catalyzed by this kinase [68]. PKC has also been linked to *N*-methyl-D-aspartate receptor (NMDAR)-mediated reduction in tau phosphorylation [69]. Overall, PKC's regulation of cytoskeleton dynamics in the brain plays a critical role not only in synapse formation and maintenance, but also in the functions of non-neuronal cells such as endothelial and glial cells [49].

### Regulation of neurotransmission proteins

PKC has long been implicated in the regulation of neurotransmission and synaptic plasticity by phosphorylating transporters, ion channels, and G protein-coupled receptors. For example, PKC phosphorylates and regulates the dopamine transporter, α-amino-3-hydroxy-5-methyl-5-isoxazolepropionic acid (AMPA)-type glutamate receptors (AMPARs), NMDA-type glutamate receptors (NMDARs), γ-aminobutyric acid (GABA) receptors, μ-opioid receptor, and metabotropic glutamate receptor 5 (mGluR5) receptors, to name a few [70–78]. It is PKC's role in postsynaptic signaling that places it in a key position to regulate synaptic plasticity; PKC phosphorylation of GluA2 subunits of AMPARs is required for AMPAR internalization from the postsynaptic membrane, thus promoting long-term depression (LTD) [79,80]. The PDZ-domain containing scaffold PICK1 mediates this internalization [80,81]. On the other hand, PKC phosphorylation of GluA1 has been implicated in the synaptic incorporation of AMPAR, thus promoting long-term potentiation (LTP) [79]. It is noteworthy that the regulation of surface receptors is a general mechanism by which PKC suppresses signaling in non-neuronal systems and is one of the mechanisms by which it functions as a tumor suppressor (see [11]). Given the importance of the dynamic regulation of receptor levels and functioning in the context of learning and memory, this function of conventional and novel PKC isozymes warrants further study, especially in the context of brain pathologies.

## PKC in the pathology of neurodegeneration

The balance between phosphorylation and dephosphorylation finely tunes the signaling output in the cell, and any changes in the normal activity of a phosphatase or a kinase can have pathological consequences. Perhaps one of the most pressing degenerative diseases of our time is the neurodegenerative AD, especially given the progressive aging of our population and the current lack of therapies for AD [82].

### PKC, amyloid β, and tau

Mounting evidence points to a key role of PKC signaling in the pathology of AD, a degenerative disease characterized by loss of synapses and plasticity mechanisms in the brain. The disease is associated with the appearance of extracellular amyloid plaques caused by the mis-cleavage of amyloid precursor protein (APP) and intracellular neurofibrillary tangles composed of hyperphosphorylated tau protein [83,84], two pathologies for which PKC involvement has been implicated over the years. But the critical importance of deregulated PKC signaling in AD was recently cemented by the results of an unbiased and comprehensive phosphoproteomic analysis of both human AD postmortem brains and brains from four AD mouse models [85]: PKC substrates accounted for over half of the core molecules that displayed increased phosphorylation in AD compared with control brains. The most robust increase in phosphorylation in AD compared with control brains occurred on MARCKS but also included PKC substrates such as GAP43. Furthermore, increases in MARCKS phosphorylation relative to other proteins occurred most significantly at early disease stages, leading the authors to propose that increased phosphorylation of this key PKC substrate initiates synapse pathology. Consistent with enhanced PKC output in AD, increased PKC levels have been implicated in AD, with early studies reporting increased staining of PKC at neurite plaques from human postmortem AD brains, including increased PKCα in reactive astrocytes associated with plaques [86,87].

One mechanism by which PKC could promote the pathology of AD is by regulating the processing of APP and production of amyloid β (Aβ) peptides [88]. However, conflicting results have been presented as to whether PKC enhances or inhibits Aβ production [89–92]. This could arise from specific PKC isozymes having unique functions, and also the use of phorbol esters to probe PKC involvement; the paradoxical effect of short-term activation followed by long-term down-regulation led to the confusion in the cancer field as to their function. It is noteworthy that Aβ production requires the dynamic endocytic recycling of APP from the cell surface, and it has been found that enlargement of early endosomes is one of the earliest events in AD [93–95]. MARCKS protein, whose phosphorylation is significantly enhanced in AD, connects PKC to both of these processes; PKC phosphorylation of MARCKS causes the liberation of both PIP_2_ and filamentous actin at the plasma membrane, both of which may promote increased endocytosis into early endosomes [96].

Another mechanism by which enhanced PKC signaling promotes the pathology of AD involves the PDZ-scaffolded conventional isozyme, PKCα. Electrophysiological studies have revealed that the biological effects of Aβ at synapses are abolished in brain tissue from mice lacking PKCα or a PDZ domain scaffold it binds, PICK1 [12,97]. Synaptic depression is also prevented by treatment of rat brain slices with a PKC inhibitor (BisIV) that works on PKC bound to protein scaffolds, but not with an inhibitor (Gö6976) that does not inhibit PKC bound to protein scaffolds [98]. Aβ-induced synaptic depression can be restored by re-expression of PKCα, but not a construct lacking the PDZ ligand. These results suggest that the activity of PKCα, specifically, bound to a PDZ domain scaffold, transduces the effects of Aβ on synapses. One possible mechanism for the downstream effects of PKC could be by controlling receptor function: exposure of synapses to Aβ peptide *in vitro* causes a decrease in GluA2-containing AMPA receptors facing the synapse, and GluA2 mutants incapable of regulating endocytosis are insensitive to Aβ-induced synaptic depression [99]. PKCα and its scaffold PICK1 play a critical role in AMPAR internalization, and both PICK1 and PKCα are required for Aβ-mediated synaptic depression [12,97]. Thus, a reasonable hypothesis is that PKCα promotes neurodegeneration by removing AMPA receptors from synapses. It is also noteworthy that PKCα has been shown to be necessary for cerebellar LTD by a mechanism that depends on its intact PDZ ligand [100].

### Gain-of-function PKC mutations identified in neurodegenerative diseases

While loss-of-function somatic mutations in PKC isozymes are associated with cancer, germline gain-of-function mutations in PKC have been identified in two neurodegenerative diseases. First, activating mutations in PKCγ are causal in spinocerebellar ataxia, a progressive and often fatal degenerative disease [101,102]. Over 20 such mutations have been identified in spinocerebellar ataxia Type 14 (SCA14), many of them occurring in the C1B domain [103]. These mutations enhance the ‘open’ and signaling competent conformation of PKC.

Genome-wide sequencing of Alzheimer families resulted in the recent identification of three highly penetrant PKCα mutations that co-segregated with AD in human patients [12]. All three mutations enhanced PKCα signaling output, supporting the hypothesis that enhanced PKCα signaling may contribute to AD pathology. This is in contrast with PKC mutations identified in cancer, which all either had no effect on PKC activity or were inactivating [11]. This is perhaps unsurprising given the long-standing inverse relationship between cancer, a disease of cell proliferation, and neurodegeneration, a disease of cell death. Many of the same signaling pathways are deregulated in both diseases [104,105], and a recent meta-analysis of nine independent studies found that AD patients exhibit a 45% decreased risk of cancer compared with the general population [106].

## Directions for the field

With the onset of advanced large-scale genome sequencing technologies, the identification of disease-associated variants of PKC, its regulators, and its targets, will provide a minefield of information on how PKC signaling pathways contribute to neuronal signaling and pathologies [107]. Similarly, unbiased approaches to interrogate the transcriptome, phosphoproteome, and interactome, among other data sets, are likely to define clear signaling pathways mediated by PKC. Coupled with the development of advanced molecular, cellular, and pharmacological tools and methodologies to probe the activity and regulation of PKC within live cells [33,108,109], the coming years will likely see major advances in our understanding of PKC function in the brain. Thus, a combination of unbiased screens, large-scale genome-wide association study (GWAS), and hypothesis-driven biochemical work to support the proteomic and genomic findings poise the field to identify PKC-dependent pathways that are deregulated in neuropathologies, paving the road for new therapeutic strategies for the treatment of neurodegeneration.

## Abbreviations

Aβ: amyloid β
AD: Alzheimer's disease
AMPAR: α-amino-3-hydroxy-5-methyl-5-isoxazolepropionic acid receptor
APP: Amyloid Precursor Protein
CHO: Chinese hamster ovary
DG: diacylglycerol
GABA: γ-aminobutyric acid
GAP43: growth-associated protein 43
GSK-3β: glycogen synthase kinase-3β
IP_3_: inositol trisphosphate
LTD: long-term depression
LTP: long-term potentiation
MARCKS: myristoylated alanine-rich C-kinase substrate
mGluR5: metabotropic glutamate receptor 5
NMDAR: *N*-methyl-D-aspartate receptor
PICK1: protein interacting with C kinase 1
PIP_2_: phosphatidylinositol 4,5-bisphosphate
PKC: protein kinase C
PKM: protein kinase M
PLC: phospholipase C
PS: phosphatidylserine
PSD95: postsynaptic density protein 95
SAP97: synapse-associated protein 97
